# Eight Weeks of Plyometric Training Improves Ability to Change Direction and Dynamic Postural Control in Female Basketball Players

**DOI:** 10.3389/fphys.2019.00726

**Published:** 2019-06-13

**Authors:** Yosser Cherni, Mohamed Chedly Jlid, Hammami Mehrez, Roy J. Shephard, Thierry Paillard, Mohamed Souhaiel Chelly, Souhail Hermassi

**Affiliations:** ^1^Research Unit (UR17JS01) Sport Performance, Health & Society, Higher Institute of Sport and Physical Education of Ksar Saîd, University of La Manouba, Tunis, Tunisia; ^2^Higher Institute of Sport and Physical Education of Ksar Said, University of La Manouba, Tunis, Tunisia; ^3^Faculty of Kinesiology and Physical Education, University of Toronto, Toronto, ON, Canada; ^4^Laboratory of Movement, Balance, Performance and Health, Department of Physical Activity and Sports Sciences, University of Pau and Pays de l’Adour, Tarbes, France; ^5^Sport Science Program, College of Arts and Sciences, Qatar University, Doha, Qatar

**Keywords:** balance, change of direction, hamstring/quadriceps ratio, stretch-shortening cycle, team sports

## Abstract

The aim of this study was to examine the effects of 8 weeks of plyometric training on the ability to change direction and postural control in female basketball players. 25 national level female basketball players aged 18–27 years participated in the study. Volunteers were randomly assigned to an experimental group (*n* = 13) who replaced a part of their standard regimen by plyometric training twice weekly for 8 weeks, and a control group (*n* = 12) who continued their usual in-season training program. Before and after the intervention, the ability to change direction and postural control were assessed by force platform under both static and dynamic conditions (with the eyes open and then closed). Isokinetic testing was also performed to calculate the Hamstring/Quadriceps (H/Q) strength ratio. The intervention improved ability to change direction (*p* ≤ 0.001, *d* = 1.51) and shortened path length (*p* = 0.038, *d* = 0.937) during static balance testing. However, it did not yield significant inter-group differences in postural control in the antero-posterior plane. The stance in the medio-lateral plane seemed the most responsive to the intervention, with reductions in surface area (*p* = 0.012, *d* = 0.285), velocity with the eyes closed (*p* = 0.031, *d* = 0.968), and path length with the eyes open (*p* = 0.029, *d* = 0.968). The intervention did not change the H/Q ratio at the two speeds tested (60° and 120°.s^–1^). In summary, the addition of 8 weeks plyometric training to the usual in-season basketball regimen of top-level female basketball players enhanced their ability to change direction and reduced the risk of falls and injuries by improving postural control, but did not increase the H/Q measure of knee stability.

## Introduction

Basketball is a team sport that combines a large proportion of high intensity activities such as running, sprinting, accelerating, jumping and landing, interspersed with frequent and abrupt changes of direction, decelerations, and stops. Much of the data concerning this sport was originally collected on male players. Details of movement patterns are now accumulating for female participants, but more information is still needed on training techniques that will develop the needed physiological abilities in women’s teams.

During games, adult females make 579–1750 changes of activity as often as every 2 s (McInnes et al., 1995; Matthew and Delextrat, 2009), with players reacting to specific game situations as they anticipate the movements of an opponent (Sheppard and Young, 2006; Sekulic et al., 2017). In contrast to some sports, basketball is multidirectional, with actions required even in the frontal plane, especially during defensive “shuffles” (Ben Abdelkrim et al., 2007). Lateral shuffling occurs up to 450 times per game, with mean values of 63–298 for adult females (Taylor et al., 2017). The total distances covered are 203–269 m in adult males (Scanlan et al., 2011) and 125 m in adult females (Scanlan et al., 2012). Lateral movements occupy 18 – 42% of game time, with individual movements requiring 0.6–1.4 s of high intensity activity (Matthew and Delextrat, 2009), executed at an appropriate time, height, and speed (Gamble, 2011). Players thus need a combination of strength, power and change of direction ability in their lower limbs to win a running or jumping duel, and grasp the ball before an opponent. Nonetheless, due to the rigor of the rules, because of limited playing space, and the exigencies of the game, players are also frequently exposed to situations that upset their balance; they must control their body position and maintain balance when jumping, pivoting, shuffling, changing direction, and withstanding contact from an opponent (Plisk, 2008). Poor postural control predisposes to falls and injuries and is a critical component of common motor skills (Burke-Doe et al., 2008; Gabbard, 2011). Paradoxically, basketball players have shown a poor balance compared with participants in other sports (football, gymnastics, and swimming) (Bressel et al., 2007; Hrysomallis, 2011). A substantial overload is placed on the lower limbs, with relatively high injury rates (7–10 injuries per 1000 games), and 58–66% affecting the lower extremity (Taylor et al., 2015). Furthermore, female basketball players are particularly vulnerable, with injury rates 2–4 times higher than in male players (Malinzak et al., 2001; Bahr and Krosshaug, 2005; Taylor et al., 2015). Compared to men, women are reported to perform side-step pivoting with increased knee extension, increased knee valgus (Bahr and Krosshaug, 2005), increased quadriceps activation, and decreased hamstrings activation, and supported by altered muscle-timing patterns (Malinzak et al., 2001). However, there is as yet little information on injury prevention in female players (Taylor et al., 2015).

The vulnerability of female players may reflect poor postural control (McGuine et al., 2000; Zazulak et al., 2007) and/or an imbalance of the lower limbs muscles (Knapik et al., 1991; Pontaga, 2004), as indicated by the isokinetic peak torque ratio for hamstring and quadriceps muscles (H/Q ratio) (Soderman et al., 2001; Wilkerson et al., 2004). Deficits in hamstring strength relative to the quadriceps are associated with anterior cruciate ligament injuries, whereas larger H/Q ratios increase the tolerance of vertical ground reaction forces (Wilkerson et al., 2004).

In male players, plyometric training has been shown to enhance change of direction abilities, vertical jumping, and short sprint performances (Hammami et al., 2019), and also to increases balance, joint awareness and overall proprioception (Myer et al., 2006; Arazi and Asadi, 2011; Singh Bal et al., 2011). The relationship between plyometric training and balance has been attributed to the promotion of anticipatory postural adjustments, particularly in peripheral joints. Repeated exposure to balance and stability challenges results in appropriate feed-forward adjustments prior to landing (Asadi et al., 2015). Plyometric training may also increase the H/Q ratio (Hewett et al., 1996; Wilkerson et al., 2004; Myer et al., 2006).

The aim of the present investigation was thus to examine the effect of replacing a part of normal basketball practice by an 8 weeks of in-season plyometric training, looking at the impact upon physical qualities such as change of direction ability, postural control, and the H/Q ratio relative to controls who continued with their standard basketball training regimen.

## Materials and Methods

### Participants

The maximum number of players per team is limited at the senior level of female basketball, so we selected study participants from two clubs, both competing at the national level, and having a similar ranking. Both clubs undertook identical training sessions and had identical schedules. Twenty-six national level players aged 18–27 years volunteered for the study. They were screened for any medical or orthopedic concern that would limit participation and were assigned to either an experimental group (E; *n* = 13) or a control group who continued to follow the standard in-season training regimen (C; *n* = 13). Throughout the intervention and until the end of season no anterior cruciate ligament injuries were sustained in either subject group. However, a player from the control group did develop a quadriceps muscle tear during training, reducing the number of controls from 13 to 12 (C; *n* = 12). The age and anthropometric characteristics of the two groups are presented in Table 1.

**TABLE 1:** Initial characteristics of experimental and control groups.

	**Experimental group (*n* = 13)**	**Control group (*n* = 12)**
Age (years)	20.9 ± 2.6	21.0 ± 3
Height (m)	1.72 ± 0.06	1.73 ± 7.24
Body mass (kg)	65.1 ± 8.8	67.3 ± 10.6
BMI (kg/m^2^)	21.9 ± 2.1	22.5 ± 2.7
Years of basketball practice	10.8 ± 3.2	10.8 ± 4.8

*n, number of subjects; BMI, body mass index.*

Participants had already achieved a good overall physical condition at the beginning of the season (having completed 4 training sessions per week for 2 months). They had subsequently followed regular training and had agreed not to change or increase their exercise habits during the course of the intervention.

### Experimental Design

The study was approved by the Manouba University Ethics Committee. After being informed about the nature, risks and benefits of the study, volunteers signed their informed consent in accordance with the Declaration of Helsinki. They were assured that they could withdraw from the trial without penalty at any time.

The study was conducted mid-season, over an 8-week period, from February to April. Performance data for both experimental and control groups were collected prior to the intervention and 5 days after the experimental subjects had completed the supplementary plyometric training.

### Training Program

All subjects normally trained 4–5 times a week and participated in an official match every Saturday, avoiding any additional training not associated with the basketball team. A test familiarization session was undertaken 3 days prior to the intervention, to minimize learning effects. During the 8-week intervention, members of the experimental group replaced a part of their usual in-season regimen with a plyometric program; this was undertaken twice per week, with a 48 h interval between sessions. The plyometric supplement followed the recommended protocol to improve performance with a minimal risk of injury (Slimani et al., 2016). Unilateral and bilateral jump drills, including horizontal and vertical jumps, were carried out in the sagittal plane, on a stable surface. In terms of training intensity, volume, and height of jumps, we followed the principle of progressive overload, starting with lower intensity, single-joint, and less complex exercise techniques, and progressing to higher intensities, multi-joint exercises, and more complex techniques (Table 2). Athletes were verbally encouraged to maximum effort without knee valgus, with a focus on improving the efficiency and power of jumps.

**TABLE 2:** Plyometric training program.

**Weeks**	**Exercises**	**Sets**	**Reps**	**Total ground contacts per session**
1–2	Bounding jumps	4	6	72
	0.4-m hurdle jumps	4	6	
	0.4-m drop jumps	4	6	
3–4	Bounding jumps	5	6	90
	0.4-m hurdle jumps	5	6	
	0.4-m drop jumps	5	6	
5–6	Bounding jumps	6	6	108
	0.5-m hurdle jumps	6	6	
	0.5-m drop jumps	6	6	
7–8	Bounding jumps	7	6	126
	0.5-m hurdle jumps	7	6	
	0.5-m drop jumps	7	6	

*Reps, repetitions.*

#### Test Conditions

None of the subjects had prior exposure to the tests of postural control and dynamic balance, hence the familiarization sessions. Participants were fully hydrated when tested, but they abstained from caffeine-containing beverages for 4 h, and from food for 2 h before testing. They also had an appropriate night of sleep prior to definitive initial and final tests, which were carried out at the same time of day (09:00–12:00 am), and under the same environmental conditions (20–25°C).

#### Anthropometrics

The body height and mass were assessed using a stadiometer and weighing scales, respectively.

#### Ability to Change Direction *T*-Test

**FIGURE 1:**
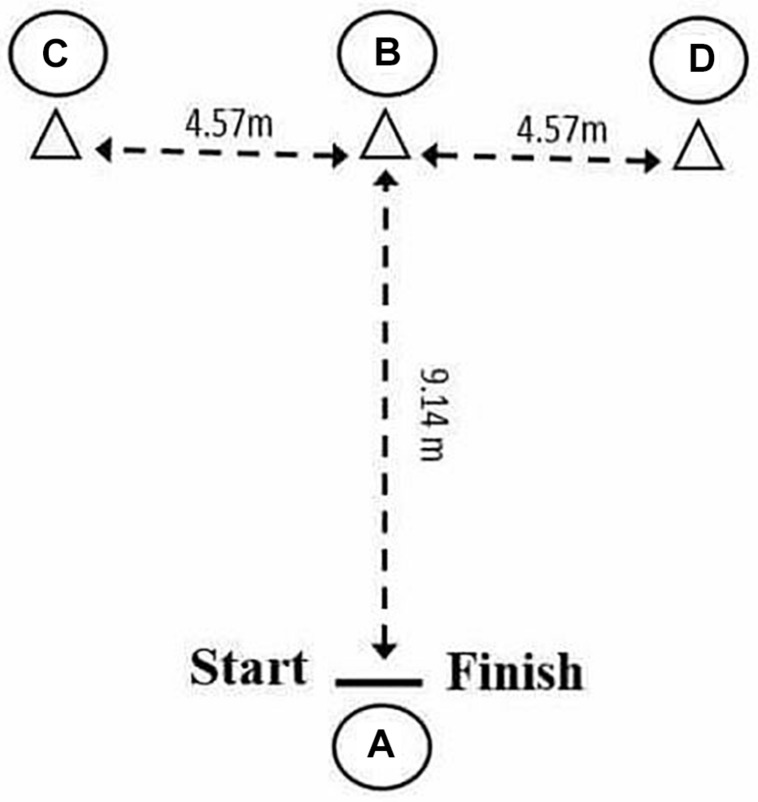
*T*-test scheme.

Ability to change direction was measured using a standard *T*-test (Semenick, 1990). Four cones were set in the form of a “T.” Cones A and B were set 9.14 m apart. The remaining two cones C and D were placed 4.57 m on either side of cone B. Before testing, subjects completed a 15-min warm-up that included jogging, lateral displacements, dynamic stretching, and light jumping. The definitive test began from cone A, with the subject sprinting to cone B and touching its base with her right hand. She then moved left, shuffling sideways to touch the base of cone C with her left hand. She then shuffled to the right, touching the base of cone D with her right hand, shuffled back to cone B touching it with her left hand, and finally ran backward to cone A (Figure 1). All movement times were recorded using an electronic timing gate (Microgate SARL, Bolzano, Italy) mounted at a height of 0.75 m. Timing began as the subject passed through the gates, and stopped when she passed through them on her return.

#### Postural Control

Postural control was assessed using a 3 strain gauge force platform (Posture Win©, Techno Concept, Mane, France) with a sampling frequency of 40 Hz and 12 bits A/D conversion. Balance was tested with the eyes both open (EO) and closed (EC), under static conditions (stable ground), and dynamic conditions (lying on a see-saw device with a 55 cm cylinder radius and a height of 6 cm). Instability was measured in the antero-posterior and medio-lateral directions. Subjects stood barefoot, with their arms by their sides. With their eyes open, they looked at a fixed-level target (1 cm^2^) set at a distance of 2 m, and with their eyes closed, they maintained the same posture. The six postural conditions were assessed in random order.

In the static condition, the test lasted 51.2 s and in each dynamic condition, the subject maintained the seesaw platform as horizontal as possible for 25.6 s. A total loss of balance invalidated the trial.

Postural control was assessed quantitatively by measuring movement of the center of pressure (Paillard and Noé, 2015). Fitting the data with an ellipse, the surface area of foot pressure quantified 90% of the total area covered in antero-posterior and medio-lateral planes; the smaller this area, the better the individual’s postural control. The path length quantified two-dimensional displacement; again, the smaller the path length, the better the postural stability. The center of pressure velocity (the accumulated center of pressure displacements divided by time) was the third and most sensitive measure; the lower this velocity, the better the postural control. To evaluate the visual contribution, the Romberg index (QRBG) was calculated as the quotient of the surface area with the eyes closed divided by that with open eyes, multiplied by 100 [(surface EC/surface EO) × 100].

##### Isokinetic testing

Subjects performed an initial warm-up (10 min of jogging, followed by 5 min of pedaling and concluding, with stretching of the lower-extremity muscles). They then sat on the dynamometer (Biodex, Medical Systems, Inc., Shirley, NY, United States) with the hips and knees flexed to 90°, and were immobilized by a diagonal strap around the trunk, a waist strap, bilateral thigh straps, and a shin strap. They hung onto the sides of the chair with both hands, and were verbally encouraged to maximal effort. Starting with the dominant leg, tests included knee movements back and forth at 60° and 120°.s^–1^. Gravitational corrections were applied, based on the weight of the limb and accessories and a computer program calculated the hamstring/quadriceps ratio (H/Q) at peak power.

### Statistical Analyses

Statistical analyses were carried out using the SPSS 20 program for Windows (SPSS, Inc., Chicago, IL, United States). The normality of data was tested using the Shapiro-Wilk test. Descriptive data are presented as adjusted group means and standard deviations. Between-group differences at baseline were examined using independent *t*-tests, and the effect of the intervention was determined by 2-way analyses of variance with repeated measures [Experimental vs. Control and Test vs. Retest]. When there were baseline differences between groups, an analysis of covariance (ANCOVA) was run. To evaluate within-group pre-to-post performance changes, paired sample *t*-tests were applied. Effect sizes were calculated by converting partial eta-squared values to Cohen’s *d*; these were classified as small (0.00 ≤ *d* ≤ 0.49), medium (0.50 ≤ *d* ≤ 0.79), and large (*d* ≥ 0.80) (Cohen, 1988). The significance level was set at *p* < 0.05 throughout.

## Results

Initial measures showed that the majority of parameters did not differ between experimental and control groups and training-related effects were assessed using 2-way analyses of variance with repeated measures. However five measures (one parameter for static balance and four parameters for dynamic balance in the antero-posterior plane) showed initial differences and an ANCOVA was then applied. Neither group showed any significant change in anthropometric measures following the intervention. However, the experimental group speeded their ability to change direction by 4% (*p* ≤ 0.001), from 11.62 ± 0.60 s to 11.16 ± 0.48 s, whereas, times for the control group remained unchanged (11.65 ± 0.37 vs. 11.87 ± 0.60 s).

In terms of postural performance, Static stance (Table 3) showed significant intervention effects (group × time interaction) on the path length with the eyes open (Δ–14.2%, *p* = 0.038, *d* = 0.937). However, in the dynamic condition, the intervention had no significant effects in the antero-posterior plane, despite an improvement in the delta change (% Δ) for path length (EO and EC) and velocity (EO) in the experimental group compared to the controls (Table 4). In contrast, the intervention enhanced the performance of all tested variables in the medio-lateral plane (Table 5), with a significant decrease of surface area (*p* = 0.012, *d* = 0.285) and of velocity (*p* = 0.031, *d* = 0.968), and an improvement in path length (EO) (*p* = 0.029, *d* = 0.968). Results from the Romberg Index demonstrated no effect of training on the players’ visual dependence to maintain their balance under any of the three tested conditions. For the H/Q ratio, at the velocity of 60°.s^–1^, the experimental group showed significant improvements in both dominant and non-dominant legs from pretest to posttest (*p* = 0.020, *d* = −0.59; *p* = 0.042, *d* = −0.73), but values for the control group did not change. Likewise, there were no significant group by time interactions at a velocity of 120°.s^–1^ (Table 6).

**TABLE 3:** Static balance, pre-, and post-intervention.

	**Experimental group (*n* = 13)**			**Paired *t*-test**		**Control group (*n* = 12)**			**Paired *t*-test**		**ANOVA Group × Time**		**ANCOVA**	
	**Pre**	**Post**	**% Δ**	***P* value**	***d* (Cohen)**	**Pre**	**Post**	**% Δ**	***P* value**	***d* (Cohen)**	***P* value**	***d* (Cohen)**	***P* value**	***d* (Cohen)**
**Surface area (mm^2^)**														
*Eyes open*	78.2 ± 26.5	136 ± 244	50.2 ± 18.8	0.371	−0.36	70.1 ± 39.6	82.8 ± 42.8	46.8 ± 88.1	0.273	−0.32	0.498	0.285		
*Eyes closed*	112 ± 70.5	224 ± 337	149 ± 331	0.272	−0.48	133 ± 70.3	116 ± 68.8	4.15 ± 56.5	0.482	0.37	0.954	0.408		
*Rombeg Index*	143 ± 63.4	196 ± 78.7	56.8 ± 67.8	0.061	−0.77	207 ± 81.5	162 ± 86	−21.3 ± 31.5	0.061	0.56			0.071	0.41
**Path length (mm)**														
*Eyes open*	0.72 ± 0.10	0.61 ± 0.12	−14.2 ± 16.1	0.008	1.04	0.76 ± 0.21	0.81 ± 0.18	10.9 ± 30.2	0.474	−0.27	0.038	0.937		
*Eyes closed*	0.80 ± 0.22	0.75 ± 0.18	−1.85 ± 27.5	0.453	0.21	0.84 ± 0.22	0.88 ± 0.31	3.64 ± 19.4	0.554	−0.16	0.338	0.408		
**Velocity (mm.s^–1^)**														
*Eyes open*	6.80 ± 0.99	6.34 ± 1.61	−6.3 ± 21.3	0.307	−0.77	7.28 ± 1.93	7.66 ± 1.62	9.32 ± 26.7	0.500	−0.22	0.236	0.505		
*Eyes closed*	9.28 ± 2.97	9.40 ± 2.04	7.6 ± 31.5	0.890	−0.05	9.97 ± 2.69	10.20 ± 3.91	2.25 ± 20.9	0.735	−0.08	0.908	0.000		

**TABLE 4:** Dynamic balance in antero-posterior plane before and after intervention.

	**Experimental group (*n* = 13)**			**Paired *t*-test**		**Control group (*n* = 12)**			**Paired *t*-test**		**ANOVA Group × Time**		**ANCOVA**	
	**Pre**	**Post**	**% Δ**	***P* value**	***d* (Cohen)**	**Pre**	**Post**	**% Δ**	***P* value**	***d* (Cohen)**	***P* value**	***d* (Cohen)**	***P* value**	***d* (Cohen)**
**Surface area (mm^2^)**														
*Eyes open*	124 ± 51	133 ± 50	25.4 ± 73.1	0.665	−0.18	126 ± 69	143 ± 70	31.6 ± 62.8	0.450	−0.25	0.794	0.000		
*Eyes closed*	956 ± 587	1106 ± 784	44.9 ± 12.5	0.584	−0.22	1463 ± 2011	901 ± 403	11.1 ± 74.8	0.330	0.40	0.677	0.505		
*Romberg index*	807 ± 396	838 ± 496	18.8 ± 65.4	0.868	−0.07	971 ± 627	749 ± 550	8.33 ± 103	0.418	0.39			0.920	0.351
**Path length (mm)**														
*Eyes open*	0.78 ± 0.14	0.73 ± 0.14	−4.9 ± 20.4	0.255	0.37	0.90 ± 0.13	0.91 ± 0.23	0.8 ± 18.5	0.848	−0.06			0.189	0.95
*Eyes closed*	0.92 ± 0.25	0.76 ± 0.32	−12.1 ± 36.3	0.163	0.58	0.93 ± 0.47	0.92 ± 0.24	0.9 ± 29.7	0.902	−0.03	0.579	0.201		
**Velocity (mm.s^–1^)**														
*Eyes open*	15.0 ± 2.9	14.4 ± 2.2	−1.3 ± 19.4	0.498	0.23	17.4 ± 2.4	18.2 ± 4.8	4.0 ± 21.1	0.535	−0.20			0.144	1.00
*Eyes closed*	39.1 ± 11.8	36.2 ± 10.8	−1.8 ± 33.3	0.515	0.28	41.6 ± 12.5	38.3 ± 8.5	−4.5 ± 19.0	0.246	0.32			0.143	0.000

**TABLE 5:** Dynamic balance in medio-lateral plane before and after intervention.

	**Experimental group (*n* = 13)**			**Paired *t*-test**		**Control group (*n* = 12)**			**Paired *t*-test**		**ANOVA Group × Time**	
	**Pre**	**Post**	**% Δ**	***P* value**	***d* (Cohen)**	**Pre**	**Post**	**% Δ**	***P* value**	***d* (Cohen)**	***P* value**	***d* (Cohen)**
**Surface area (mm^2^)**												
*Eyes open*	170 ± 54	135 ± 56	−18.1 ± 32.7	0.051	0.67	171 ± 85	136 ± 47	−10.9 ± 38.5	0.084	0.54	0.991	0.000
*Eyes closed*	1463 ± 734	1058 ± 619	−23 ± 38	0.042	0.79	1191 ± 680	961 ± 604	−12 ± 48	0.284	0.37	0.012	0.285
*Romberg index*	858 ± 330	860 ± 515	6.8 ± 59.5	0.985	−0.01	800 ± 528	672 ± 229	9.74 ± 70.9	0.375	0.33	0.527	0.285
**Path length (mm)**												
*Eyes open*	1.06 ± 0.23	0.80 ± 0.14	−21.4 ± 12.7	0.000	1.35	1.09 ± 0.27	1.04 ± 0.22	−2.06 ± 21.9	0.439	0.21	0.029	0.968
*Eyes closed*	0.82 ± 0.33	0.84 ± 0.28	15.5 ± 58.9	0.844	−0.07	0.93 ± 0.28	1.04 ± 0.23	25.1 ± 60.3	0.197	−0.45	0.441	0.351
**Velocity (mm.s^–1^)**												
*Eyes open*	20.3 ± 4.1	15.4 ± 3.1	−22.9 ± 13.1	0.000	1.41	21.7 ± 5.7	19.8 ± 4.0	−4.96 ± 22.9	0.213	0.39	0.068	0.806
*Eyes closed*	49.6 ± 13.8	37.7 ± 9.3	−23.0 ± 12.2	0.000	1.06	46 ± 10	43.6 ± 10.7	−2.75 ± 26	0.516	0.24	0.031	0.968

**TABLE 6:** Hamstrings to quadriceps strength ratio (%).

	**Experimental group (*n* = 13)**			**Paired *t*-test**		**Control group (*n* = 12)**			**Paired *t*-test**		**ANOVA Group × Time**	
	**Pre**	**Post**	**% Δ**	***P* value**	***d* (Cohen)**	**Pre**	**Post**	**% Δ**	***P* value**	***d* (Cohen)**	***P* value**	***d* (Cohen)**
**Ratio at 60°.s^–1^ (%)**												
*Dominant*	65.7 ± 9.2	70.3 ± 7.6	7.8 ± 10.6	0.020	−0.59	61.1 ± 6.6	62.7 ± 8.3	3.13 ± 13.9	0.534	−0.22	0.506	0.201
*Non-dominant*	64.9 ± 11.7	71.8 ± 7.6	13.6 ± 21.5	0.042	−0.73	70.2 ± 15.1	69 ± 7.2	0.65 ± 16.3	0.756	0.11	0.190	0.392
**Ratio at 120°.s^–1^(%)**												
*Dominant*	76.3 ± 8.1	75.6 ± 10.5	−0.3 ± 16.1	0.827	0.08	71.1 ± 9.1	68.1 ± 6.1	−3.42 ± 10.7	0.223	0.41	0.644	0.142
*Non-dominant*	77.8 ± 8.0	75.4 ± 8.08	−2.2 ± 14.5	0.423	0.31	77.5 ± 8.6	75.0 ± 6.6	−2.55 ± 10.5	0.311	0.34	0.983	0.000

## Discussion

The aim of the present study was to test the effects of an 8-week in-season plyometric training intervention on ability to change direction and control posture in national level female basketball players. As hypothesized, the data demonstrated a significant improvement in ability to change direction and in the static and dynamic postural control, with a trend to a significant increase in H/Q ratio.

Previous authors have recommended continuation of a plyometric training program into the basketball season (Arazi and Asadi, 2011). The gains in ability to change direction observed here support such a recommendation, as do most empirical studies to date. Arazi et al. (2014) previously observed that plyometric training enhanced ability to change direction in untrained women (*t*-test *p* < 0.05; −1.1 s; Δ7.6%; ES = 1.1). Likewise, Miller et al. (2006) found that a plyometric program enhanced Illinois Agility test scores (9 males and 5 females) (4.9% for the *T* test and 2.9% for the Illinois agility test). Singh Bal et al. (2011) found increases of agility of male basketball players (aged 18–24 years) after 6 weeks of plyometric training (2 sessions a week), and Asadi and Arazi (2012) noted 9% decreases in agility times for the T test after 6 weeks (2 days/week) of high-intensity plyometric training in young male basketball players (19–20 years). The only negative report (Lehnert et al., 2013) found no significant changes in the T test scores of elite male basketball players after 6 weeks of plyometric training.

Gains in ability to change direction rapidly reflect neural adaptations such as an increased nerve conduction velocity, a reduction in time required for voluntary muscle activation, and better coordination between the central nervous system signal and proprioceptive feedback (Craig, 2004). Under-pinning perceptual components include a change of direction, anticipation, and decision-making processes, improved inter-muscular coordination (Slimani et al., 2016), and enhanced motor unit recruitment strategy (Miller et al., 2006; Asadi and Arazi, 2012; Asadi, 2013).

Our findings on balance and stability seem in line with previous studies evaluating plyometric exercise in team sports. Myer et al. (2006) showed that high school female volleyball players (aged from 14 to 17 years; 4 to 6 years of experience) decreased their medio-lateral center of pressure after 7 weeks (3 times a week) of plyometric training, despite the absence of change in the antero-posterior center of pressure. Benis et al. (2016) observed that national female basketball players (age = 20 ± 2 years) enhanced their scores on the Y balance test in both postero-medial and postero-lateral but not in the anterior plane following 8 weeks of biweekly body-weight neuromuscular training. However, Arazi and Asadi (2011) found no significant improvement in the dynamic balance of male basketball players (age = 18.8 ± 1.5 years; sport experience = 4.8 ± 2.5 years) after 8 weeks of plyometric training performed twice per week. These discrepancies could be due to the type of training applied (intensity, number of contacts, and plyometric drills), to differences in the methods of assessment of postural control and to differences in the sampled population (sex, age, and years of experience).

A decrease in the velocity of the center of pressure during dynamic control with the eyes closed was demonstrated in a study of female basketball players who had undergone balance-agility training (Zemková and Hamar, 2010); in the absence of visual cues, it seems there can be a more efficient integration of other somato-sensory signals, leading to an increase in proprioception (Zemkova and Hamar, 2010; Han et al., 2015; Paillard and Noé, 2015). Fong and Ng (2013) have suggest that regular physical activity can boost proprioception by improving the cortical representation of certain joints, and decreasing onset latencies in specifically trained muscle groups (Osborne et al., 2001). Other studies, also, have shown a positive effect of plyometric training on proprioception (Hewett et al., 1996; Potteiger et al., 1999; Arazi and Asadi, 2011).

The balance tests show that the current intervention induced a significant enhancement in surface area only in the medio-lateral plane (Eyes Open: % Δ = −18.1 ± 32.7; *p* = 0.051 and Eyes Closed: % Δ = −23.4 ± 38; *p* = 0.012) (Table 5). Nevertheless, such changes could allow players to avoid infractions of basketball rules such as walking with the ball or fouls during defensive phases of a game. The shortening of the medio-lateral path length under both static and dynamic conditions implies a decrease in the energy expended to maintain balance, and it could allow this energy to be used in other performance-related actions (Paillard and Noé, 2015) such as during free throws. Improvements of balance in the medio-lateral plane are of particular interest in basketball, since the majority of actions occur in the medio-lateral plane (Matthew and Delextrat, 2009; Taylor et al., 2017). Our results assume that there is a collateral transfer between the sagittal plyometric drills and improvements in medio-lateral postural control. However, the absence of improvement in the Romberg index may indicate a dependence of players upon visual input to maintain their balance. Players are accustomed to controlling the ball and watching the movements of their partners and opponents in order to collaborate or compete with them flawlessly.

Improvements in postural balance seem likely to reduce lower extremity injury risk (Myer et al., 2006) and reflect positive functional adaptations (Hrysomallis, 2011; Behm et al., 2015), particularly enhanced proactive and feed-forward adjustments that activate appropriate muscles before landing (Marigold and Patla, 2002; Paillard et al., 2005), and increased proprioceptive input (Paillard, 2009). The concomitant development of ability to change direction and postural control underlines the strong inter-relationship between the two abilities (Little and Williams, 2005; Miller et al., 2006; Sheppard and Young, 2006; Sekulic et al., 2013).

The eight weeks of plyometric training did not induce significant changes in H/Q ratio at either of the two speeds tested (60° and 120°.s^–1^). Pfile et al. (2016) also saw no significant changes in the H/Q ratio of women’s basketball players (age 19.4 ± 1.4 years) after a 6 week pre-season combined plyometric and strength training program undertaken 3 times per week. Fry and Powell (1987) suggested that generalized strength training may not improve the H/Q ratio, and therefore the hamstrings peak torque, unless specific hamstring exercises are incorporated into the protocol. Wilkerson et al. (2004) found gains in female collegiate basketball players (aged 19.0 ± 1.4 years) after 6 weeks of a pre-season program combining plyometric, stretching, and isotonic strengthening, with benefits evident at 60°⋅s^–1^ (*p* = 0.035) but not at 300°⋅s^–1^ (*p* = 0.253). Hewett et al. (1996) tested the effect of a 6 week plyometric and strength program on female volleyball players (age = 15.0 ± 0.6 years) and contrary to the present study, they found significant gains in the H/Q ratio (dominant leg *p* < 0.05, non-dominant leg *p* < 0.01). Age, genetics, gender, and level of physical training may all influence the training response of the H/ Q ratio (Alentorn-Geli et al., 2009). Moreover, a lack of improvement in the ratio may be due to an increase in the peak power of the quadriceps that is equal to or greater than that of the hamstrings. The absence of improvement at 120°.s^–1^ could also be due to a velocity-dependent response, since the plyometric exercises were undertaken at a velocity other than 120°. s^–1^. At 60°.s^–1^ pre- to post-test results showed an improvement in the dominant leg (% Δ = 7.8 ± 10.6), but associated with a greater increase in the non-dominant leg of the experimental group (% Δ = 13.6 ± 21.5), with little or no change in the control group. Hewett et al. (1996) recommended seeking enhanced function at 60°.s^–1^ as the minimum velocity to avoid a high risk of knee injury, and the improvement we found at 60°.s^–1^ could be considered as evidence of better balance in both dominant- and non-dominant legs and thus beneficial in terms of preventing knee injuries.

Sekulic et al. (2013) have demonstrated a significant correlation between gains in agility and increases of muscle power, especially in female athletes. This improvement in H/Q ratio (strength) observed here, although minimal, may have contributed to the improvement of postural control in the static and dynamic medio-lateral planes (Horlings et al., 2008; Melzer, 2009; Forte et al., 2014; Gomes et al., 2015).

## Limitations

An extension of the run time and the number of ground contacts made during plyometric training is recommended, with a view to improving trends and attempting to extend the significant gains in the postural control to planes other than the medio-lateral. Extension to a larger sample should also allow an assessment of the impact upon injuries. Subjects could usefully include players of both sexes, at various ages and at differing levels of playing ability. Further, consideration should be given to testing the relative effectiveness of other schedules of plyometric training that differ in their content and duration.

## Practical Applications

The present study shows the practical value of substituting a part of the usual basketball training regimen of female players by 8 weeks of in-season plyometric training. This program improves physical abilities such as change of direction ability as well as postural control in the medio-lateral plane and a trend to adjusting the balance of the knee may also reduce the risk of injuries. The present findings should help coaches and physical trainers in their quest to optimize daily training routines, and to enhance the performance of players throughout the playing season.

## Author Contributions

MSC and MJ contributed to formal analysis. YC, HM, and MJ investigated the study. SH, YC, HM, and MJ performed the methodology. MSC and YC contributed to project administration. MSC and SH supervised the study. TP, YC, and MSC wrote the original draft of the manuscript. RS, MSC, and SH wrote, reviewed, and edited the manuscript.

## Conflict of Interest Statement

The authors declare that the research was conducted in the absence of any commercial or financial relationships that could be construed as a potential conflict of interest.
